# MicroRNA Let-7 targets AMPK and impairs hepatic lipid metabolism in offspring of maternal obese pregnancies

**DOI:** 10.1038/s41598-021-88518-8

**Published:** 2021-04-26

**Authors:** Laís A. P. Simino, Carolina Panzarin, Marina F. Fontana, Thais de Fante, Murilo V. Geraldo, Letícia M. Ignácio-Souza, Marciane Milanski, Marcio A. Torsoni, Michael G. Ross, Mina Desai, Adriana S. Torsoni

**Affiliations:** 1 Laboratory of Metabolic Disorders (Labdime) – Faculty of Applied Sciences (FCA), University of Campinas (UNICAMP), 1300, Pedro Zaccaria St, Limeira, SP 13484-350 Brazil; 2 Institute of Biology (IB), University of Campinas (UNICAMP), Campinas, SP Brazil; 3 The Lundquist Institute and David Geffen School of Medicine at Harbor-UCLA Medical Center, University of California, Los Angeles, CA USA

**Keywords:** Epigenetic memory, Non-coding RNAs

## Abstract

Nutritional status during gestation may lead to a phenomenon known as metabolic programming, which can be triggered by epigenetic mechanisms. The *Let-7* family of microRNAs were one of the first to be discovered, and are closely related to metabolic processes. Bioinformatic analysis revealed that *Prkaa2*, the gene that encodes AMPK α2, is a predicted target of *Let-7*. Here we aimed to investigate whether *Let-7* has a role in AMPKα2 levels in the NAFLD development in the offspring programmed by maternal obesity. *Let-7* levels were upregulated in the liver of newborn mice from obese dams, while the levels of *Prkaa2* were downregulated. *Let-7* levels strongly correlated with serum glucose, insulin and NEFA, and in vitro treatment of AML12 with glucose and NEFA lead to higher *Let-7* expression. Transfection of *Let-7a* mimic lead to downregulation of AMPKα2 levels, while the transfection with *Let-7a* inhibitor impaired both NEFA-mediated reduction of *Prkaa2* levels and the fat accumulation driven by NEFA. The transfection of *Let-7a* inhibitor in ex-vivo liver slices from the offspring of obese dams restored phospho-AMPKα2 levels. In summary, *Let-7a* appears to regulate hepatic AMPKα2 protein levels and lead to the early hepatic metabolic disturbances in the offspring of obese dams.

## Introduction

Adverse conditions during fetal and early postnatal life can have a long-term impact on health and metabolism, a phenomenon known as the *Developmental Origins of Health and Disease* (DOHaD) or metabolic programming^1^. Maternal obesity and high-fat diet consumption have been shown to drive deleterious effects on the offspring metabolism, predisposing them to the development of obesity, insulin resistance, dyslipidemias, and non-alcoholic fatty liver disease (NAFLD)^2–6^.

NAFLD is considered as a complex interaction of nutritional factors and higher susceptibility due to parental obesity programming of epigenetic mechanisms, and microRNAs (miRNAs) may play an essential role in NAFLD development, progression and diagnostics^7,8^. *Let-7* was one of the first miRNAs to be discovered^10^, and processing and maturing of *Let-7* can be inhibited by LIN28^9^. *Let-7*/LIN28 axis is implicated in energy metabolism, and regulates multiple aspects of glucose and lipid metabolism, while *Let-7* knockdown using antimir-based approaches appears as a potential strategy for treating metabolic diseases^9,10^.

NAFLD individuals commonly have decreased hepatic AMPK^11,12^. The maintenance of AMPK levels seems to be beneficial for the liver homeostasis, and the activation of AMPK may be of value for the prevention and treatment of metabolic disorders associated with obesity, such as type 2 diabetes mellitus and NAFLD^13^.

Thus, in the present study we sought to investigate whether hepatic *Let-7* would have a role in the modulation of AMPKα2 in the context of the NAFLD development in the offspring programmed by maternal obesity during intrauterine life.

## Results

### Let-7/AMPK axis is modulated in the liver of offspring from obese dams

Offspring from obesity-prone HFD-fed dams (OP-O), unlike the offspring from obesity-resistant HFD-fed dams (OR-O), had lower body weight and higher serum glucose, insulin and NEFA (C-O) (Supplemental Fig. 1).

**Figure 1:**
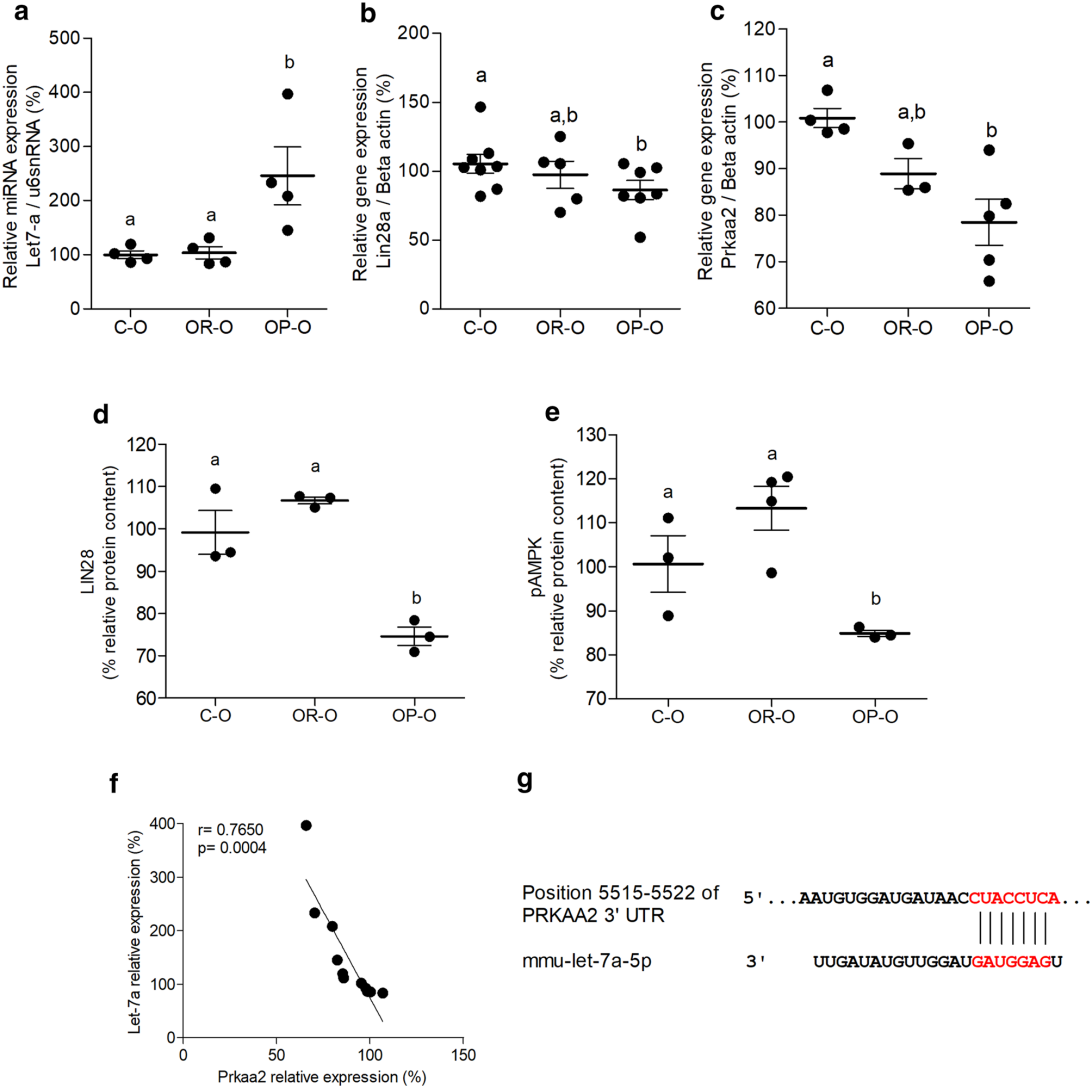
*Let-7*/AMPK potential axis is modulated in the liver of newborn male offspring from obese dams. qRT-PCR of *Let-7*a **(a)**, Lin28a **(b)**, and *Prkaa2* **(c)**, Indirect ELISA of LIN28 **(d)** and pAMPK **(e)** in the liver of male offspring from control (C–O), obesity-resistant (OR–O) and obesity-prone (OP–O) dams at the delivery day (d0) (n = 3–8 pups/group). Pearson correlation and linear regression of hepatic levels of *Let-7*a and *Prkaa2* (n = 11) **(f)**, predicted pairing of target region of *Prkaa2* mRNA (top) and *Let-7a* miRNA (bottom), according to TargetScan, release 7.2 **(g)**. One-way ANOVA were used to compare groups. Bonferroni post-hoc test was used to determine a significance level of *p* ≤ 0.05. Values are means with their standard errors represented by vertical bars. Different letters indicate statistical significance between groups.

Hepatic gene expression was modulated in male OP-O, as they had higher levels of hepatic *Let-7*a (Fig. 1a), while the levels of *Lin28a* and *Prkaa2* were downregulated (Fig. 1b,c, respectively). *Let-7a* and *Prkaa2* expression were also modulated in female OP-O (Supplemental Fig. 2). Hepatic protein content of LIN28 and pAMPK were lower in OP-O (Fig. 1d,e, respectively). Altogether, *Let-7*a and *Prkaa2* mRNA levels showed a strong negative correlation (Fig. 1f). Bioinformatic analysis revealed that the gene encoding AMPKα2 protein is a predicted target of *Let-7*a (Fig. 1g), thus we proceeded to investigate the mechanisms underlying this finding.

### Let-7 expression can be upregulated by NEFA, glucose and TNFα, and its transfection leads to the downregulation of AMPKα2

**Figure 2:**
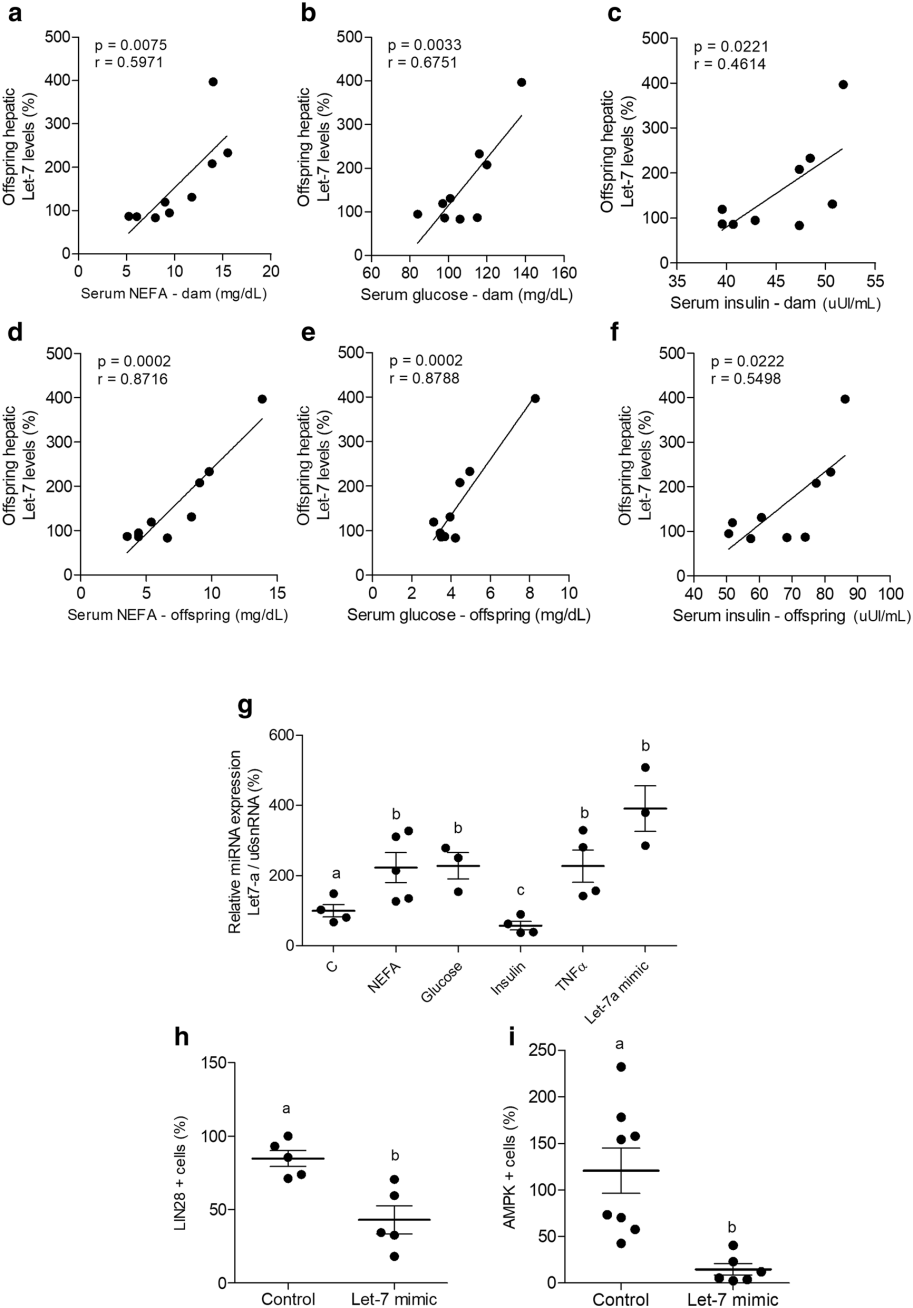
NEFA, glucose and TNFα drives *Let-7a* upregulation, which direct downregulates AMPKα2 levels. Pearson correlation of hepatic *Let-7*a in male offspring with maternal NEFA **(a)**, glucose **(b)**, and insulin **(c)**, and Pearson correlation of hepatic *Let-7*a in male offspring with offspring’s NEFA **(d)**, glucose **(e)**, and insulin **(f)** (n = 9); qRT-PCR of *Let-7*a in AML12 cell extract treated with NEFA (500 μM), glucose (20 mM), insulin (120 nM), TNFα (40 ng/mL) or *Let-7*a mimic (10 nM) for 24 h **(g)**; confocal immunofluorescence quantification of LIN28 **(h)** and AMPKα2 **(i)** positive cells of AML12 cell extract transfected with negative control or *Let-7*a mimic (10 nM) for 24 h. The correlations were constructed by using all experimental groups (control, HFD-fed prone to obesity and HFD-fed resistant to obesity). Experiments with the cell line were performed in quadruplicates and repeated twice. Student’s T test were used to compare the results at confocal immunofluorescence analysis. One-way ANOVA were used in the qRT-PCR analysis to compare all groups. Bonferroni post-hoc test was used to determine a significance level of *p* ≤ 0.05. Values are means with their standard errors represented by vertical bars. Different letters indicate statistical significance between groups.

We previously showed that obesity-prone HFD-fed dams have higher serum NEFA, glucose and insulin levels^14^. Figure 2 (a–c) show a positive correlation between maternal serum parameters and hepatic levels of *Let-7*a in the offspring. The levels of NEFA, glucose and insulin in offspring are also correlated with their hepatic expression of *Let-7*a (Fig. 2d–f, respectively).

In AML12, the treatment with NEFA, glucose, and TNFα drove an upregulation of *Let-7*a similar to that observed after the transfection with *Let-7* mimic (Fig. 2g).

The transfection of AML12 with *Let-7*a mimic (Supplemental Fig. 3) lead to a decrease in LIN28 and AMPKα2 positive cells (Fig. 2h–i).

### Let-7a anti-miR prevents fat accumulation driven by NEFA

**Figure 3:**
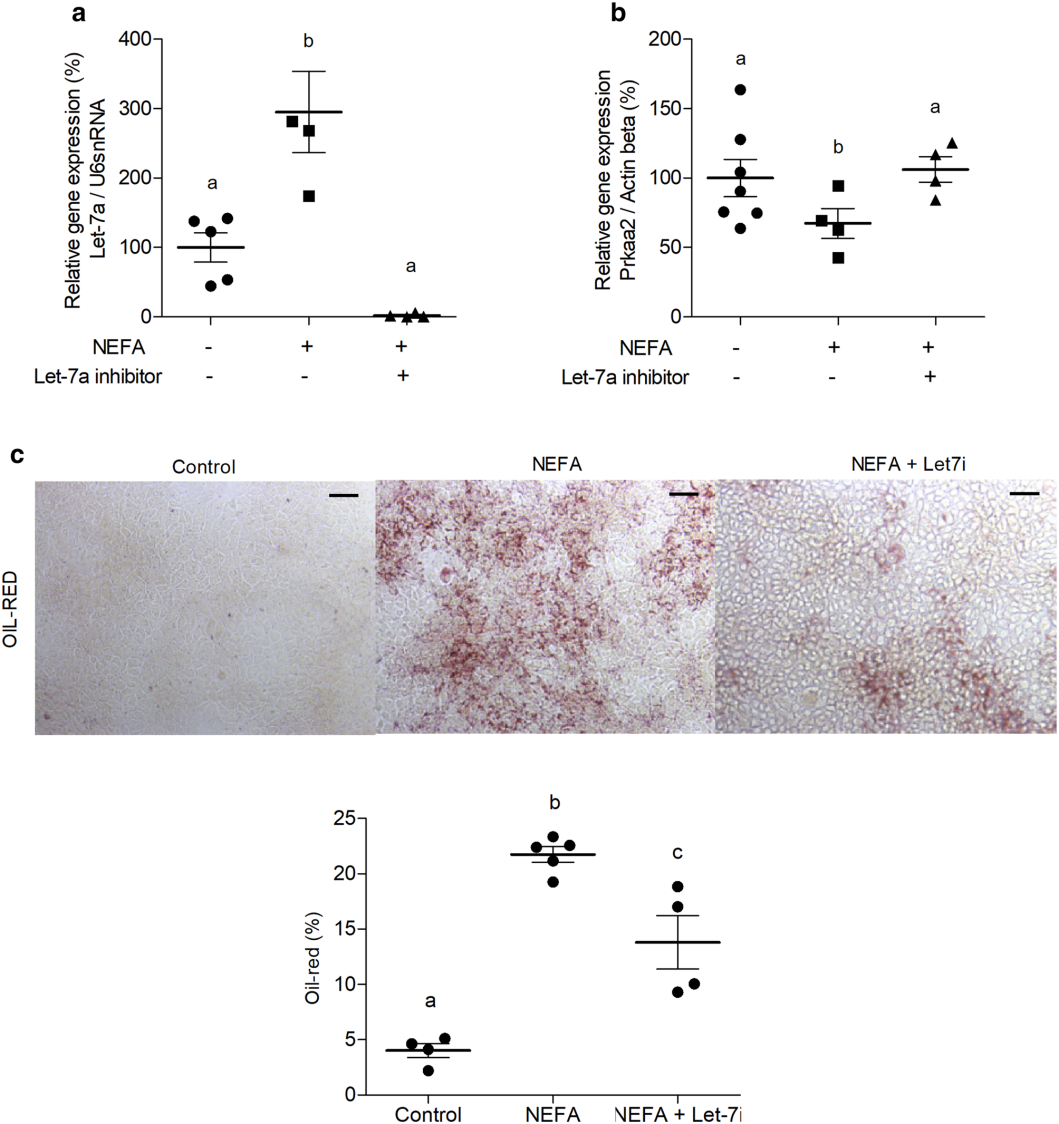
Inhibition of *Let-7a* rescues NEFA-induced alterations in hepatocytes. qRT-PCR of *Let-7*a **(a)** and Prkaa2 **(b)**; representative Oil-red images (scale-bar = 10 µm), and quantification **(c)** of AML12 cell extract treated with NEFA (500 μM) or NEFA (500 μM) plus *Let-7*a inhibitor transfection (10 nM) for 24 h. Experiments were performed in quadruplicates and repeated twice. One-way ANOVA were used in the qRT-PCR analysis to compare all groups. Bonferroni post-hoc test was used to determine a significance level of *p* ≤ 0.05. Values are means with their standard errors represented by vertical bars. Different letters indicate statistical significance between groups.

The transfection of AML12 with *Let-7*a anti-miR prior to NEFA treatment was able to impair NEFA-mediated reduction of *Prkaa2* levels (Fig. 3a,b).

The treatment with NEFA led to an increase in fat deposition, however *Let-7*a anti-miR prevented fat accumulation driven by NEFA in the hepatocytes (Fig. 3c).

### Inhibition of Let-7a rescues AMPKα2 levels in the ex-vivo liver slices of offspring from obese dams

**Figure 4:**
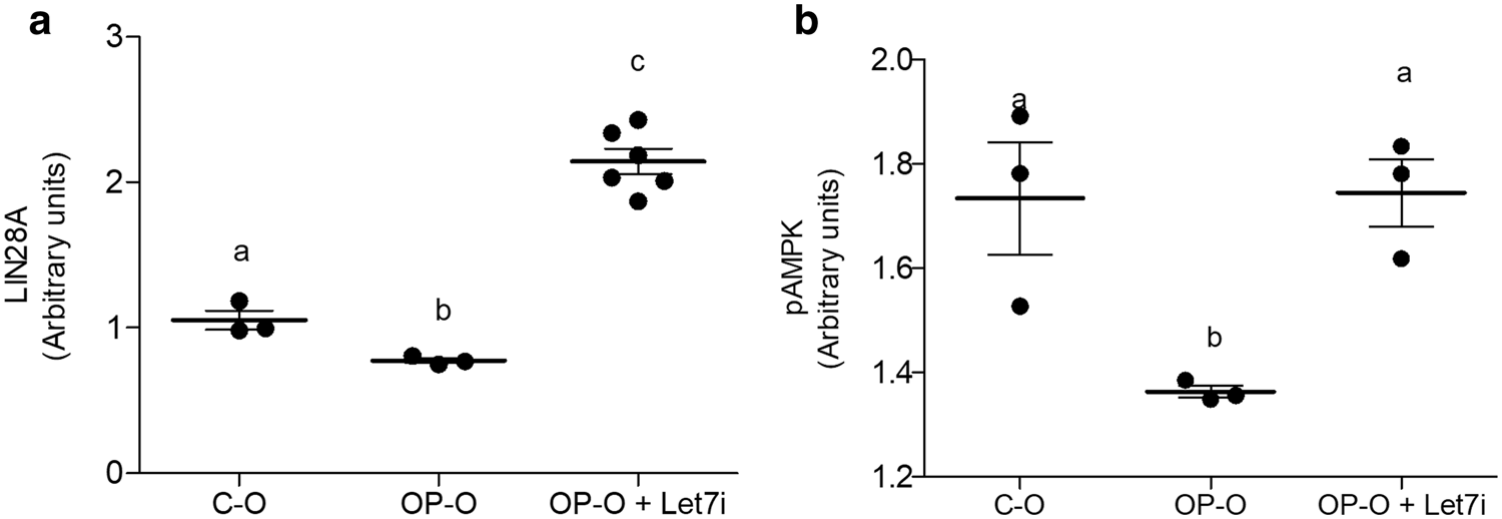
Ex-vivo manipulation of hepatic *Let-7a* rescues pAMPKα2 levels. LIN28A **(a)** and pAMPKα2 **(b)** levels of ex-vivo liver transfection of newborn male offspring with *Let-7*a inhibitor (10 nM) for 24 h by indirect ELISA. One-way ANOVA were used in the analysis to compare all groups. Values are means (n = 3–6/group) with their standard errors represented by vertical bars. Bonferroni post-hoc test was used to determine a significance level of *p* ≤ 0.05. Different letters indicate statistical significance between groups.

OP-O) had lower basal levels of LIN28, as well as phospho-AMPKα2 (Fig. 4a,b, respectively). When the liver of OP-O were transfected with *Let-7*a anti-miR, the levels of both LIN28 (Fig. 4a) and phospho-AMPKα2 (Fig. 4b) were rescued.

## Discussion

We recently showed that offspring from obesity-prone (OP-O) dams fed HFD during gestation demonstrated marked metabolic disturbances compared to offspring from obesity-resistant (OR-O) dams, with lower body weight at the delivery day (d0) being one of the main differences of OP-O from OR-O^14^. Here we showed that OP-O, but not OR-O, have higher hepatic levels of *Let-7*a and lower of Lin28a at d0. Interestingly, *Let-7* and Lin28 were firstly described as heterochronic regulators of developmental timing in *C. elegans*, and Shinoda and colleagues (2013) showed that Lin28 knockout mice exhibited dwarfism as early as in embryogenesis, and at birth they were 30–50% smaller than heterozygote controls^15^. Thus, the imbalanced levels of *Let-7*/Lin28 may, at least in part, explain the lower birth weight in offspring from obese dams.

Moreover, recent studies have shown that the balance in the *Let-7*/Lin28 axis also has a major role in the energy homeostasis, especially in alterations related to glucose and insulin signaling^10,16^. On the other hand, AMPK is known as a cellular energy sensor, and its expression and activation have been largely studied as key mechanisms to prevent and treat metabolic abnormalities related to glucose and lipid homeostasis^11,12^. We found no reports that have linked the AMPK levels with the *Let-7* modulation in the context of lipid homeostasis and NAFLD development. However, in 2012, McCarty speculated that metformin could act as an antagonist of Lin28, thus leading to *Let-7* upregulation^17^. Zhong and colleagues (2016) explored the molecular mechanisms underlying the antitumorigenic properties of metformin and they identified that the treatment of cancer cells with both metformin and AICAR, AMPK-activating agent, drove an upregulation of *Let-7* levels^18^. However, it is possible that there may be a feedback regulation among AMPK and *Let-7* levels. We identified *Prkaa2*, the gene that encodes the α2 subunit of AMPK protein, as a predicted target of *Let-7* family by computational analysis of miRNA/mRNA interaction. The inversely correlated expression of *Let-7*a and *Prkaa2* in the liver of mice acutely and chronically exposed to HFD is another evidence of their interaction.

Male newborns from obese dams had lower *Prkaa2* transcript levels and AMPKα2 proteins levels in the liver. At normal conditions, AMPK is activated by high levels of AMP, and triggers catabolic while inhibiting anabolic processes to restore cellular energy homeostasis^13^. In the liver, the activation of AMPK blocks the synthesis of fatty acids, TG, cholesterol, and proteins while activating oxidative processes^13,19,20^, and it has been shown that obese, diabetic, or non-alcoholic fatty liver disease (NAFLD) individuals have decreased hepatic AMPK^11,12^. We previously reported that male OP-O have higher hepatic TG content, and upregulated *Srebf1* expression at birth, while they present higher hepatic TG and cholesterol levels, and upregulation in *Fasn* and *Srebf1* expression after weaning^14^. These findings are consistent with the lower *Prkaa2*/AMPKα2 levels in the liver. We showed here that *Let-7* anti-miR transfection in hepatocytes can prevent fat accumulation induced by NEFA. This is consistent with the study from Frost and Olson (2011) which showed that mice fed a HFD but treated with a *Let-7* inhibithor prevented excessive fat storage in the liver^9^. In another study the constitutive expression of *Let-7* was sufficient to induce ectopic lipid accumulation in the liver^21^. Thus, the disruption in the hepatic lipid homeostasis in the offspring of obese dams may be driven by *Let-7*-induced AMPKα2 depletion.

Curiously, offspring from HFD females that did not develop the obese phenotype (obesity-resistant) were somehow protected from major metabolic disturbances and had no alterations in hepatic *Let-7*/Lin28 axis. Therefore, we hypothesized that there might be some metabolic particularity in obesity-prone dams that led to the modulation of the hepatic *Let-7* in their offspring. Accordingly, we found that some serum parameters of the dams, such as NEFA, glucose and insulin, positively correlate with hepatic levels of *Let-7*a of the offspring. Indeed, cultured hepatocytes revealed that NEFA, glucose, and TNFα treatments lead to an upregulation of *Let-7*a levels, although the correlation with insulin levels has not been observed. Katayama and colleagues (2015) have shown that *Let-7* levels can be directly regulated by glucose and TNFα in HEK293 cells, while insulin was unable to activate the *Let-7* promoter region^22^.

Despite TNFα had been able to drive *Let-7* upregulation, there is a lack of consensus as to whether that maternal cytokines can be transported to the fetus during pregnancy. Glucose and NEFA, on the other hand, have specific transporters in the placenta, GLUTs and FATPs, respectively, and are also the major energy substrates for fetal development^23,24^. Thus, we believe that the adverse conditions of obese pregnant dams, e.g. the excessive serum glucose and NEFA, may be related to the hepatic upregulation of *Let-7* and to the consequent metabolic disturbances of the offspring.

Metformin, an antidiabetic drug that have been effectively used to treat not only diabetes but related conditions, such as body weight management and NAFLD, functions primarily by activating AMPK^25^. Interestingly, here we showed that inhibition of *Let-7* may exert similar effects, leading to AMPK activation, since the *ex-vivo* transfection with *Let-7*a anti-miR rescued LIN28 and phospho-AMPKα2 levels in the liver of the newborn offspring of obese dams. Further studies are necessary in order to investigate the translational impact of the present results. However, based on our findings we believe that *Let-7* anti-miR may exert a potential therapy to prevent the fetal metabolic programming effects from obese mothers.

In summary, our data showed strong evidence that *Let-7*a may regulate hepatic AMPKα2 protein levels. Furthermore, we suggest that maternal obesity, but not maternal HFD apart from the obese phenotype, leads to hepatic modulation of the potential *Let-7*/AMPK axis, and may be related to the metabolic disturbances presented by the newborn offspring from obese dams.

## Methods

### Experimental animals and diets

All of the experimental procedures were performed in accordance with the ARRIVE guidelines, and the guidelines of the Brazilian Society of Science in Laboratory Animals and were approved by the local Ethics Committee for Animal Use (ID protocols 4349-1, and 3963-1) of the University of Campinas (UNICAMP). All experimental animals were obtained from Animal Breeding Center at the University of Campinas (CEMIB) and were maintained in individual polypropylene micro-isolators at 22 ± 1 °C and lights on from 06:00 to 18:00 h.

Twelve five-week old female Swiss mice (*Mus musculus*) were fed a standard chow diet (Nuvilab CR-1, Nuvital, PR—Brazil, C; 3.5 kcal/g, 9.5% fat) or a high-fat diet as previously described^5^ (HFD: 4.6 kcal/g, 45% fat) for an adaptation period of 4 weeks before mating. At the end of the adaptation period, HFD females were classified as obesity-prone or obesity-resistant, as described by Simino et al., 2020^14^, and they were mated with control male mice. One male for two female mice were housed together to mate. During pregnancy, female mice were fed the same diet of the adaptation period. At the delivery day (d0), newborns were euthanized and liver was dissected and immediately sectioned to *ex-vivo* analysis or frozen in liquid N_2_ followed by -80 °C storage to qPCR and immunofluorescence.

### In silico analysis of miRNA potential targets

The Let-7/mRNAs target prediction was performed using MiRWalk 2.0 platform (http://www.umm.uni-heidelberg.de/apps/zmf/mirwalk/), accessing a total of 12 algorithms. Interactions were considered valid when predicted by TargetScan algorithm, and at least 5 other algorithms.

### Quantitative real time PCR (qPCR)

Total RNA and microRNA were extracted from liver (~ 150 mg) or cells using RNAzol RT (Molecular Research Center, MRC, Cincinnati, OH—USA) according to the manufacturer’s recommendations, and quantified using NanoDrop ND-2000. Reverse transcription was performed with 3 μg of total RNA or miRNA by specific reverse transcription kits (Thermo Fisher Scientific, Waltham, Massachusetts—USA). The relative expression of mRNAs (Prkaa2 ID Mm01264789_m1, Lin28a ID Mm00524077_m1) and microRNAs (Let-7a ID 000377, U6srRNA ID 001973) was determined using a Taqman detection system (Thermo Fisher Scientific, Waltham, Massachusetts—USA). qPCR was performed on an ABI Prism 7500 Fast platform, and data were expressed as relative values determined by the comparative threshold cycle (Ct) method (2 − ΔΔCt).

### Immunofluorescence

Liver fragments from newborn male offspring was embedded in Tissue-Tek (Sakura, Torrance, CA—USA), frozen and sectioned into 12-µm-thick sections. AML12 cells were plated in round slides and treated as described below. Liver slices and AML12 cells were blocked with 3% albumin for 120 min. After, they were incubated with specific primary antibodies (AMPKα2 (1:50 dilution), Santa Cruz Biotechnology, CA—USA, or LIN28 (1:250 dilution), Abcam, Cambridge, MA—USA) overnight and secondary antibodies (Donkey anti-mouse FITC conjugated (1:250 dilution), Abcam, Cambridge, MA—USA). IMMU-Mount medium with DAPI was used to cover the slides (Vectashield, Vector Laboratories, Burlingame, CA—USA). Slices were visualized and captured by TCS SP5 II Leica confocal microscopy (Leica Microsystems, Wetzlar, Hesse—Germany). The number of AMPKα2^+^ and LIN28^+^ cells was counted using ImageJ software.

### Cell culture and transfection

In vitro analysis were performed using AML12 mouse hepatocyte cell line (ATCC CRL-2254). Cells were maintained in DMEM:HAM-F12 medium (1:1, 3.15 g/L glucose) (Sigma Aldrich), with 10% FBS, 100U/mL penicillin, 0.1 mg/mL streptomycin, 0.005 mg/ml insulin, 0.005 mg/ml transferrin, 5 ng/ml de selenium and 40 ng/mL dexamethasone, and incubated at 37 °C in 5% CO_2_. Experiments were performed between passages 10 and 20. Cells were grown as monolayers and after seeding they were treated with glucose (20 mM), insulin (120 nM), TNFα (40 ng/mL), non-esterified fatty-acids (NEFA—500 μM), or transfected with *Let-7a* mimic (10 nM, Ambion) and Lipofectamine RNAimax (Invitrogen), in serum free culture medium, for 24 h. Cells were harvested to qPCR analysis as described above. Next, cells were seeded above round slides and transfected with *Let-7a* mimic (10 nM, Ambion) and Lipofectamine RNAimax (Invitrogen), in serum free culture medium, for 24 h. Slides were submitted to immunofluorescence analysis as described above. Further, reverse transfection of *Let-7a* anti-miR (10 nM, Ambion) and Lipofectamine RNAimax (Invitrogen) were performed and 24 h after seeding, cells were treated with NEFA (500 μM) for 24 h. Cells were then harvested for qPCR analysis or Oil-Red (Sigma Aldrich) staining, as described by Mehlem et al. (2013)^26^.

### Ex vivo* analysis*

The liver of the offspring from control and obese dams (C-O and OP-O, respectively) were extracted at the delivery day, manually sectioned into ~ 2 mm slices and immediately incubated in Krebs–Henseleit buffer (KHB, 5 mM NaCl, 118 mM KCl, 1.1 mM MgSO4·7H2O, 1.2 mM KH2PO4, 25 mM NaHCO3, 2.5 mM CaCl2·2H2O, 25 mM D-Glucose, 9 mM HEPES) at 4 °C. After, slices were transferred to culture plates with high-glucose DMEM medium (Sigma Aldrich) with 100U/mL penicillin, 0.1 mg/mL streptomycin, and incubated at 37 °C in 5% CO_2_. After 2 h, slices were transferred to new plates with culture medium and negative control or *Let-7a* anti-miR (20 nM, Ambion) and Lipofectamine RNAimax (Invitrogen). After 24 h, slices were harvested to indirect ELISA analysis of phospho-AMPKα2 (1:50, Santa Cruz Biotechnology, CA – USA) and LIN28 (1:250, Abcam, Cambridge, MA—USA).

### Statistical analysis

Results are expressed as means and their standard errors. Student’s T test was used to compare two groups. Analysis of variance (ANOVA) was assessed for multiple comparisons, and Bonferroni’s post-test was used to determine the significance level of *p* ≤ 0.05.

## Supplementary Information

Supplementary Information
