# Supplementary material for: MicroRNA Let-7 targets AMPK and impairs hepatic lipid metabolism in offspring of maternal obese pregnancies
Source: Sci Rep. 2021 Apr 26;11:8980. doi: 10.1038/s41598-021-88518-8 (PMC8076304; doi:10.1038/s41598-021-88518-8)
Supplement: Supplementary file 1 — Supplementary Information [file 41598_2021_88518_MOESM1_ESM.pdf]

MicroRNA Let-7 targets AMPK and impairs the hepatic lipid metabolism in offspring of maternal obese pregnancies

Laís AP Simino [1*], *Carolina Panzarin C* [1], *Marina F Fontana* [1], *Thais de Fante* [1], *Murilo V Geraldo* [2], *Letícia M Ignácio-Souza* [1], *Marciane Milanski* [1], *Marcio A Torsoni* [1], *Michael G Ross* [3], *Mina Desai* [3], *Adriana S Torsoni* [1]

[1] Laboratory of Metabolic Disorders (Labdime) – Faculty of Applied Sciences (FCA) of the University of Campinas (UNICAMP), Limeira/SP – Brazil.

[2] Institute of Biology (IB) of the University of Campinas (UNICAMP), Campinas/SP – Brazil.

[3] The Lundquist Institute and David Geffen School of Medicine at Harbor-UCLA Medical Center, University of California, Los Angeles/CA – USA.

* **Corresponding author:** Laís AP Simino

Affiliation: Laboratory of Metabolic Disorders, School of Applied Sciences –University of Campinas

Address: 1300, Pedro Zaccaria St, Limeira, São Paulo, Brazil. Zip Code: 13484-350.

Telephone: +55-19-99123-2338.

E-mail: lsimino@unicamp.br

SUPPLEMENTAL FIGURE 1

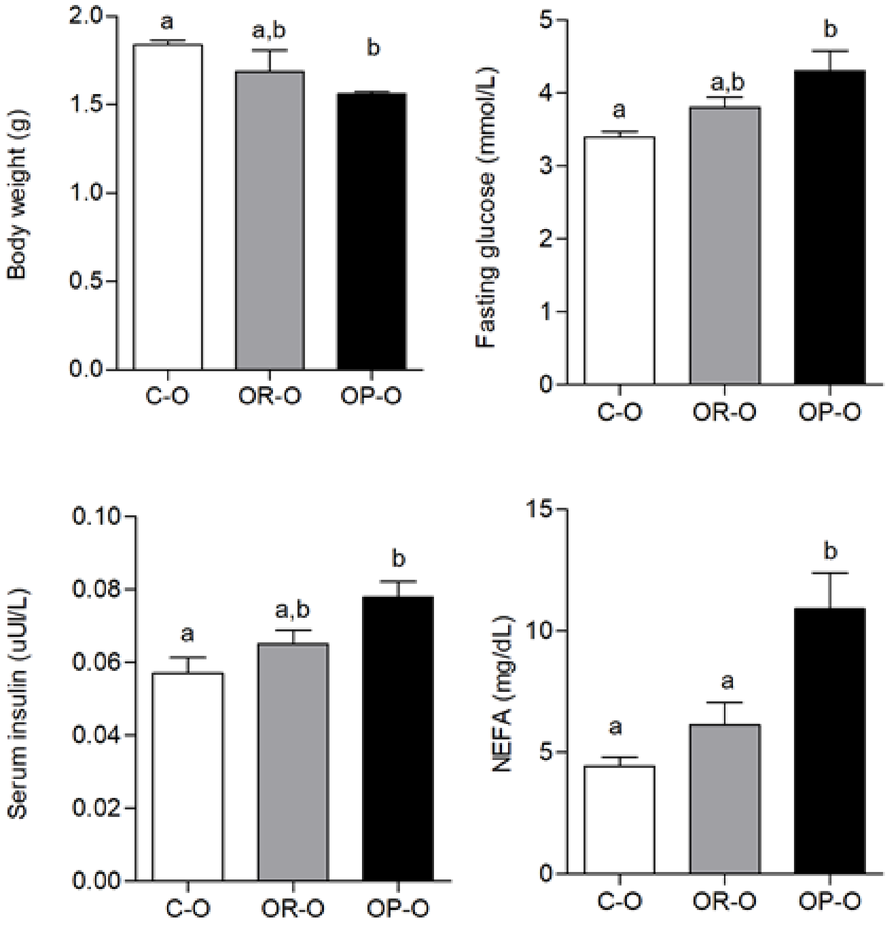

Phenotypic characteristics of offspring from control (C-O), obesity-resistant (OR-O) and obesity-prone (OP-O) dams at the delivery day.

SUPPLEMENTAL FIGURE 2

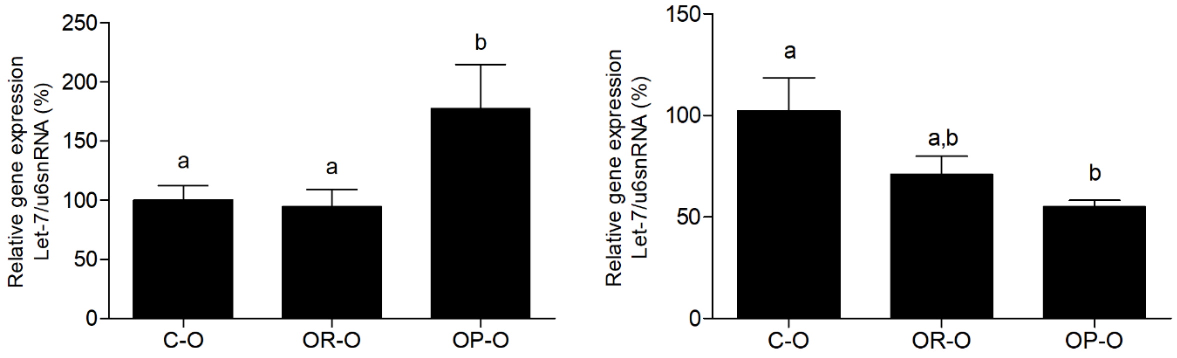

Relative gene expression (%) of Let-7a and Prkaa2 in the liver of female offspring from control (C-O), obesity-resistant (OR-O) and obesity-prone (OP-O) dams at the delivery day.

SUPPLEMENTAL FIGURE 3

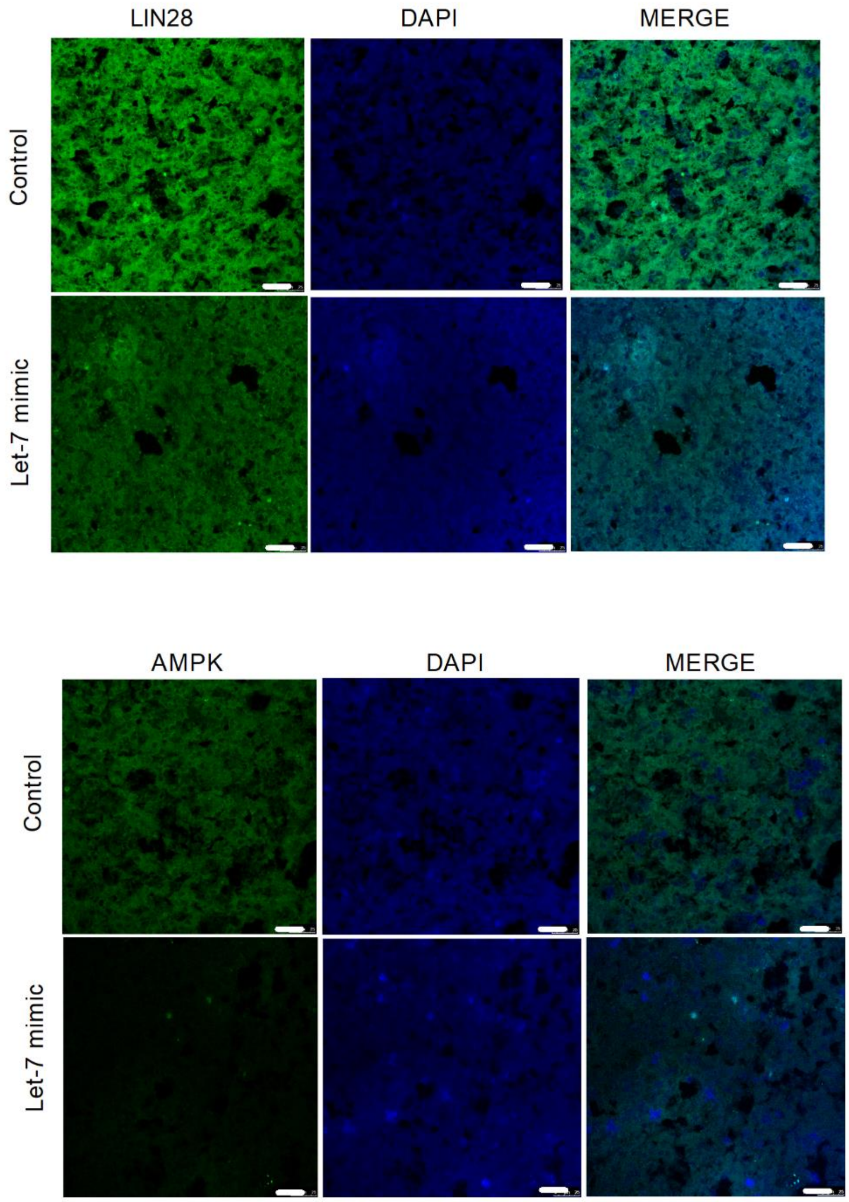

Immunofluorescence of LIN28 and AMPK levels in AML12 cells transfected with Let-7a mimic or control. Scale bars = 25µm
